# PLoS Medicine

**DOI:** 10.1371/journal.pbio.0020063

**Published:** 2004-02-17

**Authors:** Barbara Cohen

## Abstract

Announcing the next top-tier open-access journal from the Public Library of Science

Open access is gaining momentum. Authors are submitting papers in ever-increasing numbers to open-access journals. Several prominent research sponsors, including the Wellcome Trust, the Max Planck Society, the Centre National de la Recherche Scientifique (CNRS), and the Institut National de la Santé et de la Recherche (INSERM), have recently pronounced that open access is the best way for the researchers they support to publish their work. Several established commercial and not-for-profit publishers have announced plans to experiment with open-access models for some or all of their journals.

Delighted and encouraged, we gear up for the launch of *PLoS Medicine* this autumn—the next step in our efforts to bring the benefits of open access to the entire scientific and medical community. We aim to make *PLoS Medicine* a premier journal, providing open access to the best medical research to researchers, to physicians and other caregivers, and to the public.

The case for open access to medical research is even stronger than it is for basic research in biology. There are more interested parties: patients and their advocates; biotechnology and pharmaceutical companies that develop drugs and medical devices; doctors, nurses, and other healthcare providers; and health policy-makers at the national and international levels. The goal of the medical research enterprise is—or should be—scientifically, ethically, and socially responsible medicine, which means research that will benefit patients worldwide.

The reality looks somewhat different. Large investments into basic research have not yet lived up to their full potential to save lives and improve their quality. Doctors, patients, and their advocates do not have ready access to the combined peer-reviewed evidence from medical research. The prices for the latest drugs often put them out of reach of patients in poor countries or poor patients in countries without universal healthcare systems. Moreover, research focuses disproportionately on the potentially lucrative treatments for diseases of wealthy societies, shortchanging the poorer countries, which bear the greatest burden of disease.

A medical journal by itself cannot change this reality. But with the help of researchers and practicing physicians around the world who recognize the need and opportunity for change, we seek to create a journal that promotes medical research and practice that is both scientifically rigorous and compassionate.

Open access to this literature will strengthen the medical research community by giving *all* stakeholders free and immediate access to the latest medical research, along with new and more powerful search tools and links between the literature and other relevant information.

*PLoS Medicine* will be an international, modern, general medical journal, covering all areas in the medical sciences, from basic studies to large clinical trials and cost-effectiveness analyses. We will concentrate on human studies that enhance our understanding of disease epidemiology, etiology, and physiology; the development of prognostic and diagnostic technologies; and trials that test the efficacy of specific interventions and those that compare different treatments. We will publish original research and commentary that promotes translation both of basic research into clinical investigation and of clinical evidence into practice.

A truly broad medical journal is an ambitious project, but we want *PLoS Medicine* to promote an integrated understanding of the patient—to make it easy for people to read outside their specialty area. “Doctors are systems biologists,” as one medical researcher put it, and inspiration can often be found in unfamiliar territory.

Articles published in *PLoS Medicine* will be rigorously peer-reviewed. Academic and professional editors, supported by expert peer-reviewers, will select those studies that drive research forward—in this case, toward medical applications and benefits for patients.

This issue of *PLoS Biology* contains two “human” studies that met our criteria for excellence and originality, a paper by Howard Chang and colleagues (found at DOI: 10.1371/journal.pbio.0020007) on the microarray analysis of tumors and one by Sarah Rowland-Jones and coworkers (found at DOI: 10.1371/journal.pbio.0020020) that examines how HIV exhausts the capacity of the immune system. Similar papers submitted in the future will be published in *PLoS Medicine*, alongside studies that have more direct implications for clinical practice. This issue also contains several articles describing more basic advances with medical implications: a study by Terry van Dyke and colleagues (found at DOI: 10.1371/journal.pbio.0020022) describing a new mouse model for breast cancer, a report on a novel approach to drug synthesis by Chaitan Khosla and coworkers (found at DOI: 10.1371/journal.pbio.0020031), and an article by Stephen Dowdy et al. (found at DOI: 10.1371/journal.pbio.0020036) on targeted modulation of p53 activity. *PLoS Biology* will continue to solicit and publish such articles, but we will bring them—and similar ones published elsewhere—to the attention of the readers of *PLoS Medicine*.

Like *PLoS Biology*, *PLoS Medicine* must be a community journal to achieve its goals. If you are a researcher or an individual anywhere in the world who has a stake in medical research and if the goals of *PLoS Medicine* outlined here resonate with you, please contact us. *PLoS Medicine* will accept submissions beginning in April 2004, and we are looking for advocates who will help to spread the word about open access and *PLoS Medicine* in the global medical community and for investigators who will submit excellent research, review submitted articles, and contribute editorials and commentaries. *PLoS Medicine* is and will stay a work in progress, and we want to consult with as many people as possible, both before the launch and as *PLoS Medicine* evolves. Sign up to join the *PLoS Medicine* community at http://www.plos.org/medicine and help us to make the best of medical research and practice accessible to a global audience.

